# Depressive symptoms and its associated factors among prisoners in Debre Berhan prison, Ethiopia

**DOI:** 10.1371/journal.pone.0220267

**Published:** 2020-03-12

**Authors:** Yared Reta, Ruth Getachew, Melese Bahiru, Betelhem Kale, Keralem Workie, Yohannes Gebreegziabhere

**Affiliations:** 1 Program of Psychiatry, School of Nursing, Hawassa University, Hawassa, Ethiopia; 2 HIV Prevention, and Control Office, Arada Sub-city, Addis Ababa, Ethiopia; 3 Addis Ketema Sub-city Health Center, Addis Ababa, Ethiopia; 4 Ginella woreda, Ginella Health Center, Harari, Ethiopia; 5 Department of Nursing, Debre Berhan University, Debre Berhan, Ethiopia; Università degli Studi di Perugia, ITALY

## Abstract

**Background:**

Depression is a common mental disorder among prisoners characterized by a mood change involving a feeling of sadness, lack of interest, or hopelessness that lasts for weeks, months, or even longer. Besides imprisonment, depression is the primary factor leading to suicidal attempts. However, little is known about the depressive status of prisoners in Ethiopia. Therefore, this study aimed at assessing the magnitude and associated factors of depressive symptoms among prisoners of Debre Berhan prison.

**Methods:**

We conducted an institution-based cross-sectional study. We collected data from 336 randomly selected prisoners using interviewer-administered Patient Health Questionnaire-9 (PHQ-9). We collected the data from May 3 to 28, 2015, and performed binary logistic regression to identify independent predictors of depressive disorder.

**Result:**

Out of the total of 336 prisoners, 98% (n = 330) were males. Using PHQ-9 at the cut-off point of ≥5, we found the prevalence of depression to be 44% (n = 148). Being widowed (AOR = 6.30; CI: 1.09–36.67), having a college or university level educational status (AOR = 5.34; CI:1.59–17.94), having a history of suicide attempt (AOR = 2.76 CI: 1.04–7.31), having faced severe stressful life events (AOR = 2.57; CI: 1.41–4.67), being sentenced for 5 to 10 years (AOR = 2.51; CI:1.32–4.79), and having a history of chronic medical illness (AOR = 3.32 CI: 1.26–8.75) were found to be independently associated with depressive symptoms.

**Conclusion:**

There is a high prevalence of depression among prisoners of Debre Berhan prison. Hence, designing strategies for early screening and treatment of depression at prisons is crucial.

## Introduction

Worldwide, more than 10 million people are imprisoned. The prevalence of mental disorder among inmates is at least five times higher than among the general population, with clinical staff neglecting a large proportion of inmates with a severe mental illness, and that those receiving treatments do not receive adequate attention [1–3]. Once incarcerated, prisoners are no longer free to spend time and choose places to live. Deprivation of liberty because of incarceration quite often leads to a denial of choices that are usually taken for granted in the external community [4, 5].

External and internal factors worsen the morbidity of mental illness in prisons. Common external factors are overcrowding and unhygienic living conditions, poor food quality, physical or verbal abuse by prisoners, availability of illegal drugs, lack of privacy, and lack of time to relax. In addition to the stigma of incarceration, prisoners may feel guilty or ashamed of the crimes they have committed and may be concerned about the effects of their actions on others, especially their families and friends [6, 7]. The cumulative effect of all these factors increased the risk of mental illness in prisoners [8].

Depression is a significant contributor to suicide characterized by a feeling of sadness, loss of interest or pleasure, feelings of low self-esteem or guilt, disturbance of sleep or appetite, decreased energy, and poor concentration [9]. Depression can be chronic or short-lasting, markedly impairing an individual's functioning at work or school or cope with daily life. In 2015, the World Health Organization (WHO) estimated that 4.4% (322 million people) of the global population live with depression [10, 11].

Suicide is the 10th leading cause of death in the US (more common than homicide) and the third leading cause of death for ages 15 to 24 years [12]. Incarcerated individuals are more likely to attempt or commit suicide than individuals living in the general population [8]. In prison, suicide is often the leading cause of death, and over 90% of suicide deaths are caused by one or more mental disorders (commonly depression) [13, 14]. A systematic review from 12 countries revealed that prisoners were more likely to have a depressive disorder than the general population [6].

A systematic review and meta-analysis revealed an estimated 3.8% pooled prevalence of major depression among prisoners in low-and middle-income countries [15]. A study from Iran, among 351 inmates, 29% met Diagnostic and Statically Manual Four Text Revision (DSM-IV TR) criteria for a current diagnosis of major depressive disorder (MDD) [16]. In Brazil, using the Mini International Neuropsychiatric Interview (MINI) in the sample of 497 prisoners, the prevalence rate of depression in the closed and semi-open prison systems were found to be 17.6% and 18.8%, respectively [17]. In Eastern Nepal, a cross-sectional study revealed the prevalence of depression among 434 randomly selected inmates was 35.3% [18].

Coming to Africa, a study conducted in Durban, South Africa, among 193 prisoners using the Mini International Neuropsychiatric Interview (MINI), the lifetime prevalence of depression was 24.9% [7]. Similarly, an Egyptian prison study; using Psychiatric Symptoms Checklist-90 (SCL-90) revealed a high prevalence of depression (82.5%) [19]. A study in Nigeria's maximum-security prison showed the prevalence of psychiatric disorder to be 57%, and depression accounts for 30.8% [20].

In Ethiopia, a cross-sectional study in Hawassa Central Correctional Institution (n = 355) have found a prevalence of depression among prisoners to be 56.4% using the Patient Health Questionnaire (PHQ-9) [21]. Another most recent study from Bahir Dar Prison, Ethiopia, using the same tool revealed a prevalence of 45.5% [22]. A similar prevalence of depression is observed in Jimma Town Prison [23] and the prison in Northwest Amhara [22], (i.e., 41.9% and 43.8%, respectively).

Different factors may increase the probability of having depression such as gender and age; economic, educational, employment and marital status; disability and poor social support; chronic medical illness (like hypertension, epilepsy, HIV/AIDS); and history of suicide, family history of psychiatric problems and exposure to violence and crime, and acculturation stress [24–32].

Depression can cause much suffering and long-term adverse consequences. In Ethiopia, prisons are structured along with the administrative systems on which there is at least one prison at each Zone level. It is estimated that 136 prisoners per 100,000 people are found in Ethiopia, which can be considered as a large number [33]. Despite the existence of the problem, to the best of the authors' understanding, depression is hardly ever recognized and managed in prison settings of Ethiopia. Although there are similar studies in Ethiopia, the prisoners in our sample are geographically, linguistically, and culturally different from other studies. Therefore, assessing the prevalence and associated factors of depressive symptoms adds facts, reinforces other studies, and may serve as a building block to develop future intervention strategies. Hence, this study is aimed at assessing the prevalence and associated factors of depressive symptoms among prisoners of Debre Berhan prison, Ethiopia.

## Methods and materials

### Study design and setting

We used an institutional-based cross-sectional study design. The Data was collected from May 3 to 28, 2015, in Debre Berhan prison, Amhara Region, Ethiopia. Debre Berhan town, the capital of North Showa zone, is located in the North Showa zone of the Amhara regional state. It is 130 km to the north of the capital of Ethiopia (Addis Ababa). Debre Berhan town has one prison, which was established in 1948. During the study period, there were about 1,738 prisoners out of this 1704 were males, and 34 were females. Male and Female prisoners are found in prison separated by a fence. Prisoners get health care services from Debre Berhan Referral Hospital, which is located near the prison, and the prison covers the cost of health care.

### Participants and sample size

We used a single population proportion formula to calculate the sample size. We assumed the prevalence of depression to be 50% since there was no previously published research in the study setting, at a 5% margin of error and a 95% confidence level. Since our source population was less than 10,000, we have also used a correction formula. Thus, the calculated sample size was 314, and adding a 10% non-response rate; the total sample size became 345.

We selected the study participants through a simple random sampling technique, on which participants were randomly selected from the prisoner registration book by using OpenEpi Random Number Generator.

### Measurement of the study variables

Depressive symptom was the outcome variable of this study, and the exposure variables were sociodemographic and economic factors, psychological and physical health status, imprisonment status, and social support.

### Depressive symptoms

PHQ-9 was used to assess depressive symptoms. PHQ-9 is a structured questionnaire that assesses depressive symptoms, using nine of the major depressive disorder symptoms from DSM‐IV criteria. In assessing MDD, PHQ‐9 has a sensitivity of 88% and a specificity of 88% [3]. The tool is evaluated in Ethiopia and found to be reliable and valid [34–36]. PHQ-9 comprises nine items, and each item response is rated as "0" (not at all) to "3" (nearly every day), and the total score range from 0 to 27 [3]. A score of less than five represents no depression, a score of 5 to 10 is mild, 10 to 15 is moderate, 15 to 20 is moderately severe, and greater than 20 represents severe depression. For this study, a cut-off greater than or equal to five is used to classify participants as having depressive symptoms and not.

#### Sociodemographic and economic factors

We used a self-structured questionnaire with nine-items to assess the sociodemographic and economic status of the participants. The items assess information such as age, sex, marital status, ethnicity, religion, average monthly income, educational status, and occupation of the prisoners. Age was categorized into four equal age range groups with ten years of range starting from the minimum age enrolled, while economic status was categorized into five equal groups, each with 500 Ethiopian Birr (ETB) apart (1UD = 20.6 ETB, during the study period).

#### Psychological factors and imprisonment status

Fifteen self-structured items were used to assess psychological and imprisonment related factors. Psychological factors such as childhood abuse, family history of mental illness, a history of suicide attempts, and stressful life events were included in the items. While items related to the history of imprisonment include the duration of imprisonment, duration of a prison stay, and index-crime committed.

#### Physical health status

We used a questionnaire with seven self-structured items to assess the perceived physical health of the participants. The items asked about physical disability status, somatic symptoms, and other chronic diseases. To assess commonly reported somatic symptoms in people with depression, we adapted three items from the 20 items Self-Reporting Questionnaire (SRQ-20). SRQ-20 is a 20 items questionnaire aimed for assessment of mental distress, and it was previously adapted in Ethiopia and reported to be a validated screening tool [37, 38].

#### Social support

Oslo-3 item social support scale was used to assess social support with a total score ranging from 3 to 14. A score of 3 to 8 shows' poor support', 9 to 11 shows' moderate support', and 12 to 14 shows' strong support'. Previous studies reported good predictive validity for the scale regarding psychological problems [39–41]. Even, the scale was found to have acceptable validity and reliability in the African context [42].

### Data collection

We collected the data using an interviewer-administered questionnaire. Four trained health professionals collected the data, and two of the authors (YR and YG) supervised the data collection. Before the actual data collection, we pre-tested the questionnaire on 5% of prisoners at Debre Berhan town police custody. The authors, together with the data collectors, ensured completeness and consistency inflow of items to minimize systematic errors and to estimate the time needed to complete the questionnaire during the pre-test.

### Statistical analysis

We checked the data completeness and entered to EpiData entry version 3.1 and exported the data to IBM SPSS (Statistical Package for the Social Sciences) version 24 for cleaning and further analysis. We used percentages and frequencies to present descriptive statistics. Associated variables in simple logistic regression at a p-value of <0.05 were entered into the multiple logistic regression to control confounders. We reported the strength of association between the outcome variable and explanatory variables using the odds ratio with 95% CI. We used a p-value of less than 0.05 to declare a significant association with depression in the final model.

### Ethical statement

The ethical review committee of Debre Berhan University, College of Medicine and Health Sciences, reviewed the study protocol, and approved the ethical clearance (Ref No: DBUMF/216/2015). To reach the inmates, we have got a letter of permission from the Debre Berhan prison administration office. The right of the participants to refuse or discontinue participation was respected, and confidentiality was ensured. All respondents signed written informed consent before taking part in the research with no financial or other gains.

## Result

### Socio-demographic characteristics of the prisoners

Out of 345 prisoners contacted, we got complete data found from 97.39% (n = 336) prisoners. Majority (98.2% (n = 330)) of the participants were males. The age of participants ranged from 18 to 83 years, with a mean (SD) age of 28.14 (±14.25) years. Nearly two-thirds of the participants (59.2% (n = 199) were single in terms of marital status. Majority were Amhara by ethnicity (94.9% (n = 319)), and Ethiopian orthodox followers by religion (92% (n = 312)). The large proportion of the participants were employees before imprisonment (74.7% (n = 251)), had a monthly income of less than 500 ETB (72.9% (n = 245)), and had attended only primary school (57.4% (n = 193)) (Table 1).

**Table 1:** Socio-demographic characteristics of inmates of Debre Berhan prison, Ethiopia, 2015.

Variables		Frequency	%
Sex	Male	330	98.2%
	Female	6	1.8%
Age	18–24 Years	134	39.9%
	25–34 Years	134	39.9%
	35–44 Years	50	14.9%
	= >45 Years	18	5.4%
Marital status	Single	199	59.2%
	Married	115	34.2%
	Widowed	10	3.0%
	Divorced	12	3.6%
Religion	Orthodox	312	92.9%
	Protestant	4	1.2%
	Muslim	20	6.0%
Ethnicity	Amhara	319	94.9%
	Oromo	13	3.9%
	Tigray	4	1.2%
Educational status	Illiterate	52	15.5%
	Primary school	193	57.4%
	Secondary	69	20.5%
	College or University	22	6.5%
Occupation	Student	68	20.2%
	Unemployed	85	25.3%
	Farmer	64	19.0%
	Government employed	26	7.7%
	NGO	50	14.9%
	Daily labourer	31	9.2%
	Other (Retired and housewife)	12	3.6%
Average monthly income	<500birr	245	72.9%
	500-1000birr	45	13.4%
	1001-1500birr	11	3.3%
	1501-2000birr	12	3.6%
	>2000birr	23	6.8%

### Psycho-social, imprisonment and physical health status of the prisoners

About one fifth (17%, (n = 57)) of the participants were responded yes for a history of childhood abuse, and 6% (n = 20) were responded yes for the family history of mental illness. History of suicidal attempt was reported in 8.9% (n = 30) of participants, and around one-third 28% (n = 94) of the participants have passed through a stressful life event. This was their second or more imprisonment for 5.4% (n = 18) of the respondents. Homicide (47.6%, (n = 160)), and stealing and robbery (33.0%, (n = 111)) were found to be the two leading index-crimes. The mean (SD) of the current sentence was 6.5 (± 1.8) years, and the mean (SD) stay in prison was 3.4 (± 1.2) years (Table 2).

**Table 2:** Psycho-social, imprisonment and physical status of inmates of Debre Berhan prison, Ethiopia, 2015.

Variables		Frequency	%
History of childhood abuse	Yes	57	17.0%
	No	279	83.0%
Family history of mental illness	Yes	20	6.0%
	No	316	94.0%
Ever history of suicidal attempt	Yes	30	8.9%
	No	306	91.1%
Ever had a stressful life event	Yes	94	28.0%
	No	242	72.0%
Type of stress full life event	Death of beloved one	40	42.6%
	Being in prison	22	23.4%
	Money or material loss	32	34.0%
Past history of imprisonment	Yes	18	5.4%
	No	318	94.6%
Duration of the current sentence	<5 Years	109	32.4%
	5–10 Years	99	29.5%
	>10 Years	128	38.1%
Duration of current stay in prison	<5 Years	264	78.6%
	5–10 Years	72	21.4%
	>10 Years	0	0.0%
Index-crime committed	Homicide	160	47.6%
	Stealing and robbery	111	33.0%
	Rape	31	9.2%
	Abduction	19	5.7%
	Corruption	15	4.5%
Social support	Poor	266	79.2%
	Moderate	67	19.9%
	Strong	3	0.9%
Physical disability	Yes	30	8.9%
	No	306	91.1%
Types of disability	Hearing or Visual	10	33.3%
	Walking	20	66.7%
History headaches during the past one month	Yes	57	17.0%
	No	279	83.0%
History back pain during the past one month	Yes	43	12.8%
	No	293	87.2%
History of fever during the past one month	Yes	48	14.3%
	No	288	85.7%
Other health problems (HPTN, DM, Epilepsy)	Yes	35	10.4%
	No	301	89.6%

From the study participants, 8.9% (n = 30) had a physical disability. Those who often have a headache, back pain, and fever, over the past 30 days, makeup 17% (n = 57), 12.8% (n = 43), and 14.3% (n = 48) of the study participants, respectively. According to Oslo 3-item social support scale those who have poor, moderate, and strong social support accounts 79.2% (n = 266), 19.9% (n = 67), and0.9% (n = 3), respectively (Table 2).

### Prevalence of depression among prisoners

Using a cut-off point of ≥ 5 for detecting cases on PHQ-9, we found the point prevalence of depression among prisoners of Debre Berhan prison to be 44% (n = 148) (95% CI: 38.8%, 49.9%).

### Determinants of depression among prisoners of Debre Berhan prison

During Bi-variable analysis of sociodemographic characteristics, marital status, educational status, religion, and average monthly income before imprisonment were significantly associated with depression. From factors related to physical health status, the presence of headache, back pain, fever over the past 30 days, and presence of any chronic health problem were found to have a significant association with depression in the bi-variable analysis.

Multivariable binary logistic regressions were used to control confounder, and variables with a p-value of < 0.05 are reported as a significantly associated variable with depression. We find that the odds of having depression to be six times higher in widowed participants compared to those who were singles (AOR = 6.30 CI: 1.09–36.67). The odds of those participants educated at the college level or university level were five times more likely to have depression compared to participants who were illiterates (AOR = 5.34 CI: 1.59–17.94). (Table 3)

**Table 3:** Multi-variable binary logistic regression analysis of factors associated with depression among prisoners of Debre Berhan prison, Ethiopia, 2015.

				OR with 95% CI	
					Adjusted
Variables	Category	Depression		COR with 95% CI	AOR with 95% CI
		No	Yes		
Age	18–24 Years	70	64	0.35 (0.12–1.04)	1.68(0.42–6.73)
	25–34 Years	87	47	0.21 (0.07–0.62) *	0.86(0.23–3.24)
	35–44 Years	26	24	0.36 (0.11–1.15)	1.26(0.30–5.22)
	≥45 Years	5	13	R	R
Marital status	Single	117	82	R	R
	Married	64	51	1.26 (0.71–2.24)	1.43(0.80–2.57)
	Widowed	2	8	5.80(1.02–33.87) *	6.30(1.09–36.67) *
	Divorced	5	7	2.25(0.56–8.94)	1.88(0.44–7.95)
Educational status	Illiterates	33	19	R	R
	Primary school	114	79	1.20(0.64–2.27)	1.05(0.52–2.15)
	Secondary School	35	34	1.69(0.81–3.52)	1.32(0.57–3.12)
	College/ University	6	16	4.63 (1.55–13.84) *	5.34 (1.59–17.94) *
Ever sustained childhood abuse	Yes	25	32	1.8 (1.01–3.20) *	1.34(0.66–2.76)
	No	163	116	R	R
History of suicidal attempt	Yes	8	22	3.93 (1.70–9.12) **	2.76(1.04–7.31) *
	No	180	126	R	R
Ever had serious stressful life event	Yes	31	63	3.75(2.267–6.217) **	2.57(1.41–4.67) *
	No	157	85	R	R
Previously imprisoned	Yes	5	13	3.52 (1.23–10.12) *	2.94(0.84–10.29)
	No	183	135	R	R
Duration of current sentence	<5 years	71	38	R	R
	5–10 years	46	53	2.15 (1.23–3.76) *	2.51(1.32–4.79) *
	> 10 years	71	57	1.50 (0.89–2.54) *	1.04(0.55–1.95)
Headache over the past 30 days	Yes	21	36	2.556 (1.42–4.61) *	1.48(0.66–3.28)
	No	167	112	R	R
Back pain over the past 30 days	Yes	17	26	2.144 (1.12–4.12) *	0.9(0.35–2.30)
	No	171	122	R	R
Fever over the past 30 days	Yes	16	32	2.966 (1.56–5.65) *	2.34(0.99–5.53)
	No	172	116	R	R
Chronic Medical Illness	Yes	8	27	5.02 (2.21–11.42) *	3.32(1.26–8.75) *
	No	180	121	R	R

* p < 0.05

** P<0.01; R = Reference; COR = crude odds ratio, and AOR = adjusted odds ratio

The odds of having depression on those prisoners who have a history of suicide attempt were three times higher than their counterparts (AOR = 2.76 CI: 1.04–7.31); similarly, the odds of having depression is three times higher in prisoners who sustained a severe stressful life event in the past (AOR = 2.57 CI:1.41–4.67). Prisoners with a sentence of 5 to 10 years were two times more likely to have depression than those sentenced for less than five years (AOR = 2.51 CI: 1.32, 4.79) and those who have chronic medical illness also have a two times higher odds of having depression compared to those without chronic medical illness (AOR = 2.51 CI:1.32,4.79) (Table 3).

## Discussion

In this study, we found that the one-month prevalence of depression to be 44.05% (95% CI: 38.8%, 49.9%), and this finding is consistent with studies done in Ethiopia, on which a study from Jimma Prison and Bahir Dar prison reported a prevalence of 41.9% 45.5%, respectively. Both of these studies used PHQ-9 and were done in a similar study set up. These reasons can explain an agreement of both studies with our findings. A systematic review and meta-analysis in Ethiopia reported an 11% pooled prevalence of depression in the general population, which is four times less than our result [43]. This can be explained by the fact that inmates face deprivation of liberty, social isolation, and greater vulnerability to stress than the general public [6, 43].

Our study also showed a higher prevalence of depression as compared with studies from countries like Brazil (17.6%) [17] and Iran (29%) [16], the reasons might be because we used PHQ-9, which is a screening tool, while both studies used a diagnostic tool, i.e., the study from Iran used DSM-IV, and the study from Brazil used MINI. Besides, unique challenges like inadequate prison facilities that face low-income countries, overcrowding, and inadequate health services might be the reason for the high prevalence of depression among prisoners in Ethiopia.

The prevalence of depressive symptoms in our study is significantly lower than the prevalence of depression in an Egyptian prison (82.5%). The Egyptian study used a different screening tool (SCL-90), and a small sample size (n = 80) as compared to our research, and this could explain the difference in depression prevalence.

In line with previous findings from Ethiopia, our study found that the odds of having depression is higher among inmates with a history of suicide attempt [44]. Suicide is one of the common symptoms of depression or can result from the adverse effects of depression in prison [11, 45] and it is often the single most common cause of death in prisons. Therefore, any traits of suicide should be seriously considered as a psychiatric emergency [13, 14].

Consistent with the report in Malaysia [46], our study found that the odds of having depressive symptom was higher among prisoners who sustained a severe stressful life event in the past. The association between severe stressful life events and depression is supported by a finding from a systematic review [47]. This key finding may suggest the importance of strengthening the prison mental health service with psychotherapists for proper management of severe stress.

Our data showed that widowed inmates experience depressive symptoms more than singles. This finding is consistent with the study done in Northwest Amhara, Ethiopia, and a study from Rio de Janeiro, Brazil [44, 48]. Widowed are prone to depression for the reason that they could have a higher expected parental responsibility and may experience a loss of loved ones. Our study revealed that well-educated prisoners are more likely to have depressive symptoms than illiterates. In our study educated are very few in proportion (6.3%), despite being the minority, well-educated inmates may expect better treatment and respect in the prison society which they could not be granted irrespective of literacy. Such unfelt expectations and role change may lead inmates to feel depressed and feel low.

As evidenced in different studies, chronic medical illness is highly linked with depression, resulting in poor prognosis and suicide [49–51]. Our study also revealed that those prisoners who have chronic medical illnesses have higher odds of suffering from depression, and this is in line with studies from Hawassa and Jimma prisons of Ethiopia [21, 23]. Prisoners sentenced for 5 to 10 years were more likely to have depression than those sentenced for less than five years, and this finding is supported by a study from Bahir Dar prison [22]. This implies prisoners with long sentences are likely to develop depressive symptoms as compared to prisoners with a shorter sentence, and this may alarm due mental health attention for those prisoners sentenced for greater than five years in prison. In the multivariable logistic regression history of childhood abuse was not found to be associated with depressive symptoms in our study. This might be because of the limitation of our screening tool, which cannot identify the chronicity of the depressive symptoms, which mostly related to childhood abuse [52].

## Conclusion

In this study, we examined the prevalence and important predictors of depressive symptoms among prisoners of Debre Berhan prison. The result noted that a high prevalence of depressive symptoms among prisoners than depressive symptoms in the general population. Since prisons in Ethiopia are similar in terms of administrative setup and facilities, our study can be conclusive proof of the burden of depression in Ethiopian prisons.

Our statistical analysis concluded that depressive symptoms were more observed in inmates who have a history of suicide attempts, severe stressful life events, and chronic medical illness. Serving long sentences, being widowed, and well educated were variables independently associated with depressive symptoms in this study. Therefore, Debre Berhan prison administration, together with responsible governmental and non-governmental organizations need to strengthen mental health service in prisons. For example, the prison can facilitate in-service training for prison health professionals on early screening and prevention of adverse effects of depression (suicide).

Even though our study did not allow establishing a temporal relationship, supported by robust scientific evidence we concluded that prison health professionals need to give due attention for early screening and treatment of depression among inmates with a history of attempted suicide, serving a long sentence in prison and having a comorbid chronic medical illness.

## Limitations of the study

This study has some potential limitations. Since we used a cross-sectional study design, the study does not allow inferring the causation. This study was conducted in a single institution, restricted to presenting an enormous amount of information on the prevalence rate of depressive symptoms among the different populations of the Ethiopian prison. Another most important limitation of this study was that it could not assess chronic depressive symptoms that existed before imprisonment, and also stressful life event was not assessed with a standardised item. Only 6 (six) women have participated in our study, which is not sufficient to make comparisons across gender.

## Supporting information

S1 Data (SAV)

S1 File (PDF)

## Abbreviations

BDI: Beck Depression Inventory
DD: Depressive Disorder
DSM IV-TR: Diagnostic and Statically Manual Four Text Revision
DSM-5: Diagnostic and Statistical Manual of Mental Disorders, Fifth Edition
MDD: Major Depressive Disorder
MINI: Mini International Neuropsychiatric Interview
PHQ-9: Patient Health Questionnaire- 9
SCI: Structured Clinical Interview
SCL: Psychopathy Symptoms Checklist
SCL-90: Psychiatric Symptoms Checklist-90
SPSS: Statistical Package for Social sciences
WHO: World Health Organization
