# Supplementary material for: Depressive symptoms and its associated factors among prisoners in Debre Berhan prison, Ethiopia
Source: PLoS One. 2020 Mar 12;15(3):e0220267. doi: 10.1371/journal.pone.0220267 (PMC7067449; doi:10.1371/journal.pone.0220267)
Supplement: S1 File — (PDF) [file pone.0220267.s002.pdf]

IRB'28decision *Ref No: DBUMF/216/2015*

Meeting no: 12/2015 Date (D/M/Y): April DE/2015

Protocol number: 16/15/SPH. Assigned no 44

DEPRESSIVE DISORDER AND ITS ASSOCIATED FACTORS AMONG PRISONRES IN DEBRE BERHAN TOWN, NORTH SHOWA ZONE, AMHARA REGION, ETHIOPIA, 2015	
Principal investigators:	YARED RETA (MSc)
Institute:	DBU- College of Health Science
Elements reviewed(DBUMF 01-009)	☐ Attached ☑ not attached
Review of revised application<br>☑ Yes ☐ No	Date of previous review:
Decision of the meeting:	☑ approved ☐ approved with recommendation<br>☐ resubmission ☐ disapproved

I. Elements approved-
1. Protocol version no 8
2. Protocol version date 12/4/2015
3. informed consent version no 8
4. Informed consent version date 12/4/2015

II. Obligation of the pi-
1. Should comply with the standard international & national scientific and ethical guidelines
2. All amendments and changes made in protocol and consent form needs IRB approval
3. The pi should report SAE within 10 days of the event
4. End of the study, including manuscripts and thesis works should be reported to the IRB.

III. To Estimation ☐

Institution Review Board (IRB) approval: period from 12/4/2015 to 12/4/2016

Follow up report expected in

3 months___ 6 months___ 9 months_____ one year____ ,

D/Chairman, IRB
Mr. Wondossen Asegidew
Signature___
Date: 12/4/2015

Research and community service Associate dean
Mrs. Hilina Ketema
Signature___.
Date:-12/4/2015

Debre Berhan University ★ College of Health
